# *ADAM33* Gene Polymorphisms and Mortality. A Prospective Cohort Study

**DOI:** 10.1371/journal.pone.0067768

**Published:** 2013-07-04

**Authors:** Sylwia M. Figarska, Judith M. Vonk, Cleo C. van Diemen, Dirkje S. Postma, H. Marike Boezen

**Affiliations:** 1 University of Groningen, University Medical Center Groningen, Department of Epidemiology, Groningen, The Netherlands; 2 University of Groningen, University Medical Center Groningen, Department of Genetics, Groningen, The Netherlands; 3 University of Groningen, University Medical Center Groningen, Department of Pulmonology, Groningen, The Netherlands; 4 University Medical Center Groningen, Groningen Research Institute for Asthma and COPD, Groningen, The Netherlands; University of Utah, United States of America

## Abstract

The *ADAM33* gene is associated with the pathophysiology of Chronic Obstructive Pulmonary Disease (COPD) and atherosclerosis. In this study we investigated all-cause, COPD and cardiovascular mortality, in relation to single nucleotide polymorphisms (SNPs) in *ADAM33* (Q_1, S_1, S_2, T_1 and T_2) that were genotyped in 1,390 subjects from the Vlagtwedde/Vlaardingen cohort. Participants were examined at entry in 1989/1990 and followed up till evaluation of the vital status on December 31^st^, 2008. Using Cox proportional hazards regression we estimated the risk of the SNPs in relation to mortality, adjusting for gender, age, FEV_1_, height, place of residence and packyears of smoking. Additionally, we performed stratified analyses according to gender and smoking habits. After 18 years, 284 (20.4%) subjects had died (107 due to cardiovascular disease and 20 due to COPD). Individuals homozygous for the minor allele of SNP T_2 had an increased risk of all-cause and cardiovascular mortality compared to wild types: hazard ratio 3.6 (95% confidence interval 2.0 to 6.7) and 3.4 (1.2 to 9.5) respectively. Individuals homozygous for the minor allele of S_1, S_2, T_2 or Q_1 had a significantly increased risk of COPD mortality. In stratified analyses the risk of all-cause mortality associated with SNP T_2 did not change: females 3.5 (1.5 to 8.3), males 3.1 (1.2 to 7.6), never smokers 3.8 (0.9 to 16.3), ever smokers 3.6 (1.8 to 7.2). This study shows for the first time that *ADAM33* is a pleiotropic gene that is associated with all-cause, COPD and cardiovascular mortality, independent of potential confounders.

## Introduction

Human lifespan has increased over the years almost worldwide [1]. Therefore the concept of healthy ageing, defined as a high quality of life into later stages of life with an absence of age-related disease, is becoming increasingly important [2]. So far the mechanisms explaining individual differences in lifespan and susceptibility to disease are not well understood. Thirty percent of the individual variance in life expectancy is genetically determined [3], yet the specific genetic determinants of human lifespan still remain largely unknown. One of the main objectives in research on ageing is to identify people at higher risk to developing early onset pathologies commonly associated with ageing and contributing to premature death [3]. There is an unmet need for studies that increase our knowledge about determinants of the variation in human lifespan, morbidity and mortality and that highlight potential targets for prevention. One of the goals is to identify pleiotropic genes that may lead to premature death by influencing the risk of one, or more than one, disease.

A family of proteins that may be important in explaining the individual differences in lifespan is the ADAM (A Desintegrin and Metalloproteinase) family. ADAMs are membrane-anchored proteins belonging to the zinc protease superfamily [4], [5]. They play a role in cell adhesion, cell migration and proteolysis [6] and thus are fundamental to many control processes in development and homeostasis [7]. *ADAM33* might be associated with overall mortality through its link to “inflamm-ageing”. This phenomenon refers to the fact that ageing is associated with chronic, low grade inflammatory activity leading to long-term tissue damage and systemic chronic inflammation [8], which contribute to increased mortality in elderly individuals [8], [9]. ADAM proteinases can release and activate cytokines, and if a single nucleotide polymorphism (SNP) in the *ADAM33* gene would promote a pro-inflammatory or tissue damaging activity of the transcribed protein, this may contribute to early mortality events.

In 2002, Van Eerdewegh et al. identified *ADAM33* as a susceptibility gene for asthma and airway hyperresponsiveness [5]. Subsequent studies have linked polymorphisms in *ADAM33* to airway hyperresponsiveness and airway inflammation in Chronic Obstructive Pulmonary Disease (COPD), and to accelerated lung function decline and COPD development in the general population [10], [11]. Moreover, recently *ADAM33* was linked to cardiovascular disease (CVD), emphasizing its potential pleiotropic role in age-related diseases [6].

Given the physiological importance of *ADAM33* in pulmonary and cardiovascular diseases, we hypothesize that *ADAM33* has an impact on mortality due to these disorders.

The objective of the current study was to investigate whether SNPs in the *ADAM33* gene are associated with all-cause, COPD and cardiovascular mortality.

## Methods

### Ethics Statement

The study protocol was approved by the local university medical hospital ethics committee, University of Groningen, University Medical Center Groningen, The Netherlands and all participants gave their written informed consent. In 1984, the Committee on Human Subjects in Research of the University of Groningen reviewed the study and affirmed the safety of the protocol and study design.

### Study Population

We studied 1,390 subjects of the Vlagtwedde/Vlaardingen cohort participating in the last survey in 1989/1990 [11]. This general population-based cohort of white individuals of Dutch descent started in 1965 and has been followed for 25 years. Surveys were performed every 3 years, in which the Dutch version of the British Medical Council standardized questionnaire was filled in, and spirometry was performed [11]. The vital status of all participants in the Vlagtwedde/Vlaardingen study on December 31, 2008 was assessed. We evaluated three mortality outcomes, i.e. all-cause mortality (excluding external causes of death), and COPD and cardiovascular mortality (either as primary or secondary cause of death). The causes of death were coded according to the International Classification of Diseases (ICD-9 and ICD-10, Table S1). Analyses on cause specific mortality were performed at Statistics Netherlands (The Hague).

### Blood Samples and DNA Extraction

In 1989/1990 neutrophil depots from peripheral blood samples were collected and stored at –20°C. In 2003–2004 DNA was extracted from these samples with a QIAamp DNA blood mini kit (Qiagen, Hilden, Germany) and checked for purity and concentration with a NanoDrop ND-1000 UV–Vis spectrophotometer (NanoDrop Technologies, Wilmington, DE).

### SNP Selection and Genotyping

**Figure 1:**
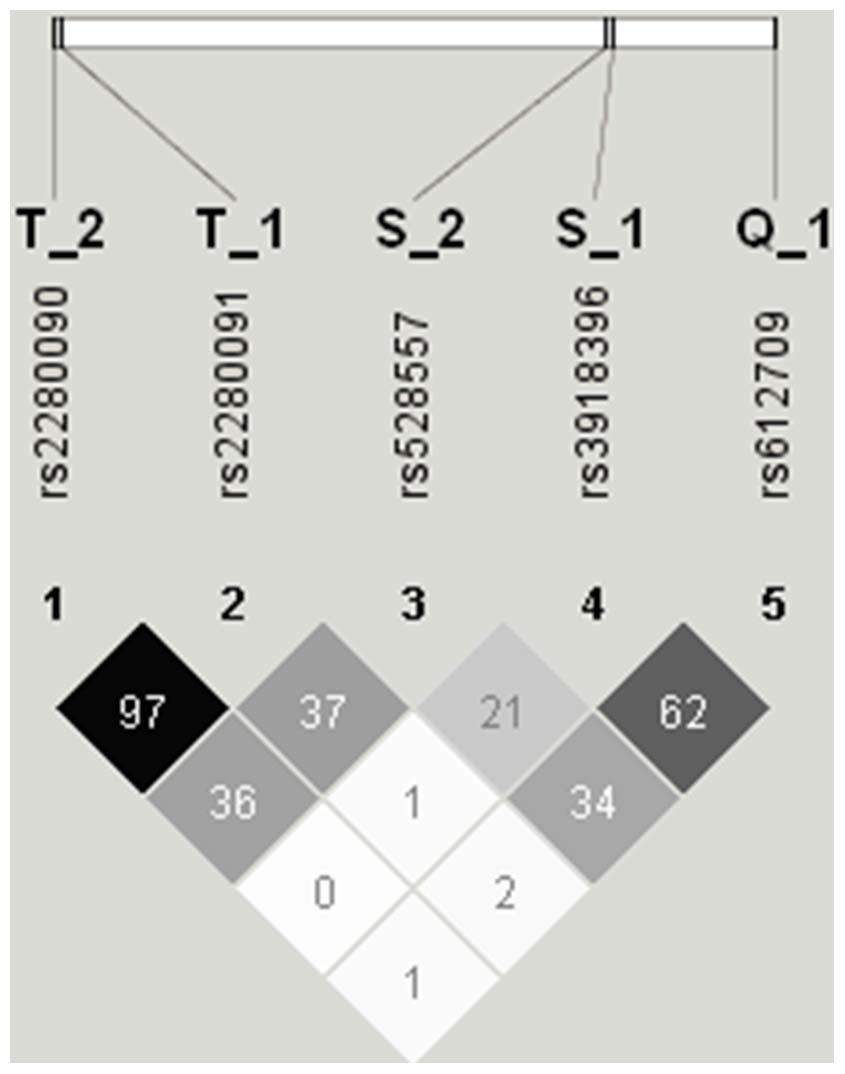
*ADAM33* linkage disequilibrium plot (100·r^2^) in the Vlagtwedde/Vlaardingen cohort.

**Figure 2:**
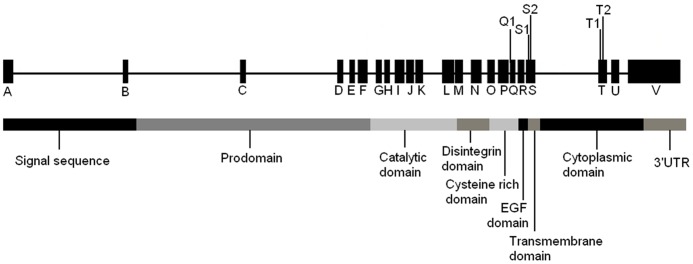
Position of genotyped SNPs in the *ADAM33* gene and the domain organization of *ADAM33* (adapted from [10]).

Five SNPs in *ADAM33*, previously linked to asthma, airway hyperresponsiveness, COPD, or accelerated decline in Forced Expiratory Volume in 1 second (FEV_1_) [5], [11] were genotyped: rs612709 (Q_1, (C/T)), rs3918396 (S_1, (Val-Ile)), rs528557 (S_2, (G/C)), rs2280091 (T_1, (Met-Thr)) and rs2280090 (T_2, (Pro-Ser)). Details on genotyping and probes for genotyped SNPs were published previously [11]. Since SNPs T_1 and T_2 are in high linkage disequilibrium (r^2^ = 0.97, Figure 1) only SNP T_2 was analyzed. Figure 2 shows the position of genotyped SNPs in the *ADAM33* gene.

### Statistical Analysis

Hardy-Weinberg Equilibrium was tested using the χ^2^ test (cut-off value p<0.05). First, descriptive analyses were performed. Differences in genotype distribution between dead and alive subjects were tested using χ^2^ tests. Cox proportional hazards regression adjusted for gender, age, FEV_1_, height, place of residence and packyears of smoking (all at the 1989/1990 survey) was used to evaluate the association between SNPs and all-cause and cause-specific (COPD and cardiovascular) mortality. Time was defined from the examination in 1989/1990 until death, end of follow-up in 2008 or last registration if subjects were lost to follow-up. Survival curves were calculated using Cox regression models. In addition, stratified analyses according to gender and smoking habits were performed.

Logistic regression adjusted for the same covariates as in the Cox regression was used to calculate odds ratios and 95% confidence intervals for the chance of survival to the age of 75 and 85 years respectively, in relation to genotypes for every SNP separately.

P-values <0.05 were considered statistically significant (tested 2-sided). All statistical analyses were performed using SPSS version 16.0 for Windows.

## Results

Table 1 shows the population characteristics at the survey in 1989/1990, according to vital status on December 31^st^, 2008. After 18 years of follow-up 78.2% (n = 1,087) of the cohort was still alive. We had an almost perfect follow-up, since only 19 (1.4%) of the genotyped participants were lost to follow-up. Among all 284 deaths, 20 (7.0%) occurred due to COPD and 107 (37.7%) due to CVD. All tested SNPs were in Hardy-Weinberg equilibrium.

**Table 1:** Characteristics of participants at visit 1989/1990 by vital status on Dec 31^st^, 2008.

Status on 31-12-2008	Alive	Dead	P value
N (%)	1,087 (78.2)	284 (20.4)	
Males	525 (48.3)	178 (62.7)	<0.001
Age	49.4 (36.0 to 72.6)	61.9 (35.8 to 79.1)	<0.001
Ever smokers	711 (65.4)	219 (77.1)	<0.001
Packyears in ever smokers	17.2 (0.1–117.1)	27.1 (0.6–262.2)	<0.001
FEV_1_, liters	2.96 (0.75)	2.48 (0.71)	<0.001
FEV_1,_ % predicted	94.6 (13.9)	84.3 (18.0)	<0.001
**Causes of death**			
COPD*		20 (7.0)	
Cardiovascular disease*		107 (37.7)	
External causes**		14 (4.9)	

All variables are expressed as number (%) or mean (SD) or median (range) as appropriate.

* Either primary or secondary cause of death, number (% of all deaths).

** Suicides, homicides, traffic accidents *etc.*

### All-cause Mortality

**Figure 3:**
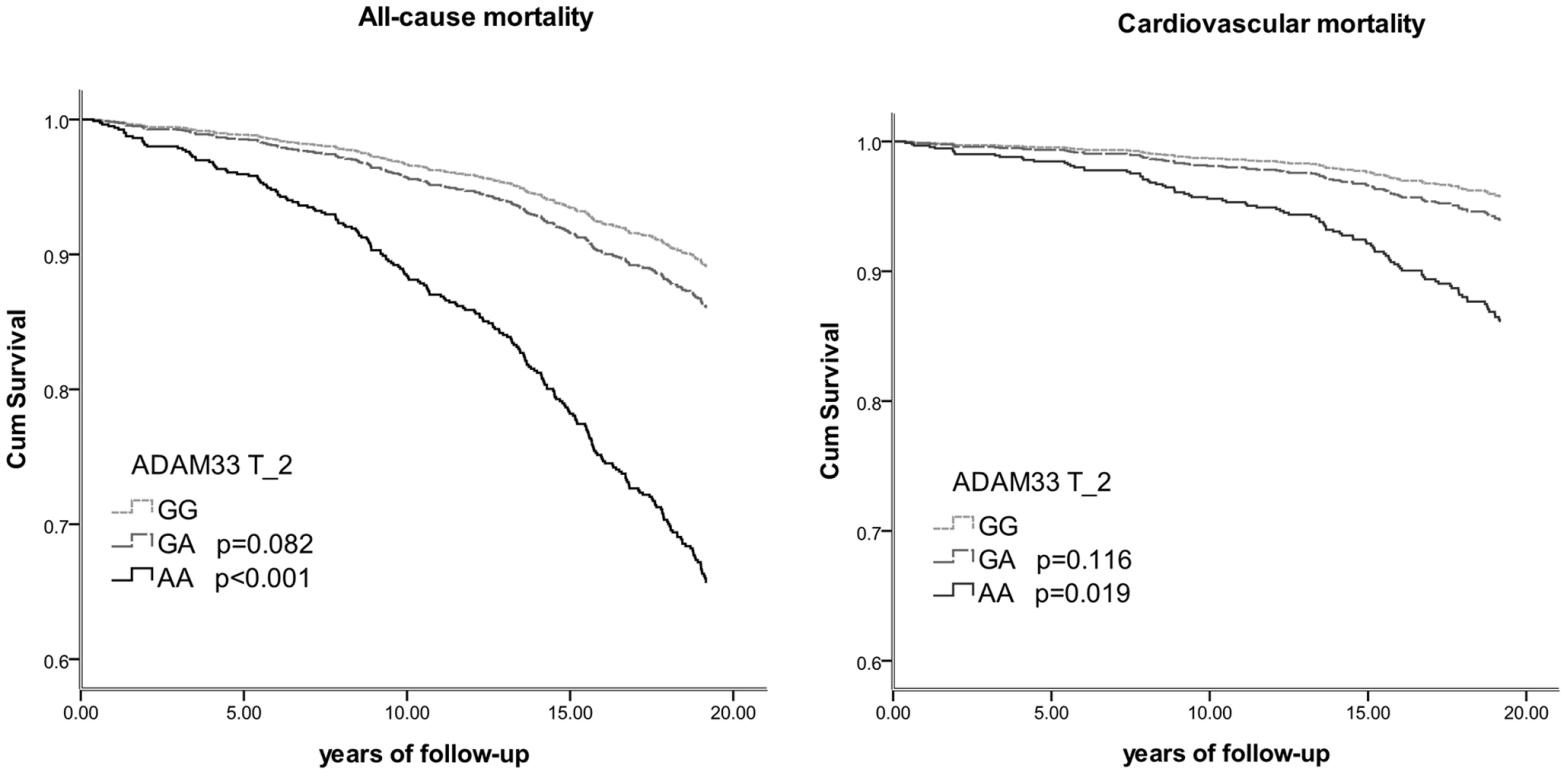
Survival curves for all-cause and CVD mortality according to SNP T_2.

Table 2 shows the genotype distributions of alive subjects and those who had died during 18 years of follow-up. The distribution of SNP T_2 was significantly different between alive and dead subjects. Furthermore, individuals homozygous for the minor allele of SNP T_2 had a significantly increased hazard ratio for all-cause mortality compared to wild types, 3.6 (95% confidence interval 2.0 to 6.7) (Table 3). SNP T_2 showed increased all-cause mortality among those with the AA genotype (Figure 3). The other investigated SNPs in *ADAM33* were not significantly associated with all-cause mortality. Table 4 presents the stratified analyses. The risk of all-cause mortality associated with SNP T_2 was similar in females (3.5, 1.5 to 8.3) and males (3.1, 1.2 to 7.6), as well as in never smokers (3.8, 0.9 to 16.3) and ever smokers (3.6, 1.8 to 7.2). Never smoking individuals, homozygous for the minor allele of SNP S_1 had a significantly increased all-cause mortality risk.

**Table 2:** Distribution of genotypes according to all-cause and cause-specific mortality.

SNP	Genotype	Alive n = 1,087	All-cause mortality n = 284	P value*	COPD mortality n = 20	P value**	CVD mortality n = 107	P value***
**Q_1**	CC	831 (77.2)	203 (77.5)		13 (65.0)		81 (78.6)	
	CT	226 (21.0)	55 (21.0)	0.965	5 (25.0)	0.024	21 (20.4)	0.822
	TT	19 (1.8)	4 (1.5)		2 (10.0)		1 (1.0)	
**S_1**	GG	913 (84.2)	228 (85.4)		12 (63.2)		91 (86.7)	
	GA	165 (15.2)	37 (13.9)	0.846	6 (31.6)	0.008	13 (12.4)	0.698
	AA	7 (0.6)	2 (0.7)		1 (5.2)		1 (0.9)	
**S_2**	GG	609 (57.3)	143 (54.2)		7 (35.0)		52 (50.5)	
	GC	386 (36.3)	99 (37.5)	0.446	9 (45.0)	0.023	44 (42.7)	0.399
	CC	68 (6.4)	22 (8.3)		4 (20.0)		7(6.8)	
**T_2**	GG	805 (76.9)	183 (69.3)		11 (57.9)		70 (67.3)	
	GA	230 (22.0)	70 (26.5)	0.001	5 (26.3)	<0.001	30 (28.9)	0.018
	AA	12 (1.1)	11 (4.2)		3 (15.8)		4 (3.8)	

* Differences between alive subjects and those who died (excluding external causes of death) tested with χ^2^ test.

** Differences between alive subjects and those who died due to COPD tested with χ^ 2^ test.

*** Differences between alive subjects and those who died due to CVD tested with χ^ 2^ test.

**Table 3:** Hazard ratio (95% CI) of all-cause, COPD and cardiovascular mortality.

SNP	Genotype	All-cause mortality*	COPD mortality**	CVD mortality**
		HR (95% CI)	HR (95% CI)	HR (95% CI)
**Q_1**	CT	1.0 (0.8–1.4)	1.6 (0.5–5.3)	1.0 (0.6–1.7)
	TT	0.7 (0.2–2.2)	7.6 (1.6–37.2)***	0.5 (0.1–3.9)
**S_1**	GA	0.8 (0.5–1.2)	2.3 (0.8–7.1)	0.7 (0.4–1.3)
	AA	1.6 (0.4–6.6)	38.4 (3.8–389.3)***	2.3 (0.3–16.9)
**S_2**	GC	1.1 (0.9–1.5)	1.4 (0.4–4.6)	1.4 (0.9–2.2)
	CC	1.6 (1.0–2.6)	6.1 (1.6–23.1)***	1.5 (0.7–3.3)
**T_2**	GA	1.3 (1.0–1.7)	0.9 (0.2–3.2)	1.4 (0.9–2.3)
	AA	3.6 (2.0–6.7)***	13.8 (3.3–58.3)***	3.4 (1.2–9.5)***

Cox regression adjusted for gender, age, FEV_1_, height, place of residence and packyears smoking (all at the last survey 1989/1990).

* Excluding external causes of death.

** Primary or secondary causes of death.

*** P value <0.05.

**Table 4:** Risk of all-cause mortality according to gender and smoking habits.

SNP	Genotype	Gender		Smoking status	
		Females	Males	Never smokers	Ever smokers
		HR (95% CI)	HR (95% CI)	HR (95% CI)	HR (95% CI)
**Q_1**	CT	1.0 (0.6–1.7)	1.0 (0.6–1.7)	1.3 (0.7–2.5)	0.9 (0.7–1.4)
	TT	1.0 (0.1–7.2)	1.0 (0.1–7.2)	1.2 (0.2–9.0)	0.6 (0.1–2.2)
**S_1**	GA	0.6 (0.3–1.2)	0.6 (0.3–1.2)	0.7 (0.3–1.7)	0.8 (0.5–1.2)
	AA	4.2 (0.6–30.7)	4.2 (0.6–30.7)	7.9 (1.0–61.4)*	0.9 (0.1–6.8)
**S_2**	GC	1.1 (0.7–1.8)	1.1 (0.7–1.8)	0.9 (0.5–1.6)	1.2 (0.9–1.7)
	CC	1.6 (0.8–3.5)	1.6 (0.8–3.5)	1.7 (0.6–4.8)	1.6 (0.9–2.7)
**T_2**	GA	1.0 (0.6–1.6)	1.0 (0.6–1.6)	0.7 (0.4–1.4)	1.5 (1.1–2.1)*
	AA	3.5 (1.5–8.4)*	3.5 (1.5–8.4)*	3.8 (0.9–16.3)**	3.6 (1.8–7.1)*

**Females** n = 676 (103 deaths); **Males** n = 714 (166 deaths); **Never smokers** n = 445 (62 deaths); **Ever smokers** n = 945 (207 deaths); n = 14 deaths due to external causes are excluded.

* P value <0.05.

** P = 0.07.

### COPD Mortality

**Figure 4:**
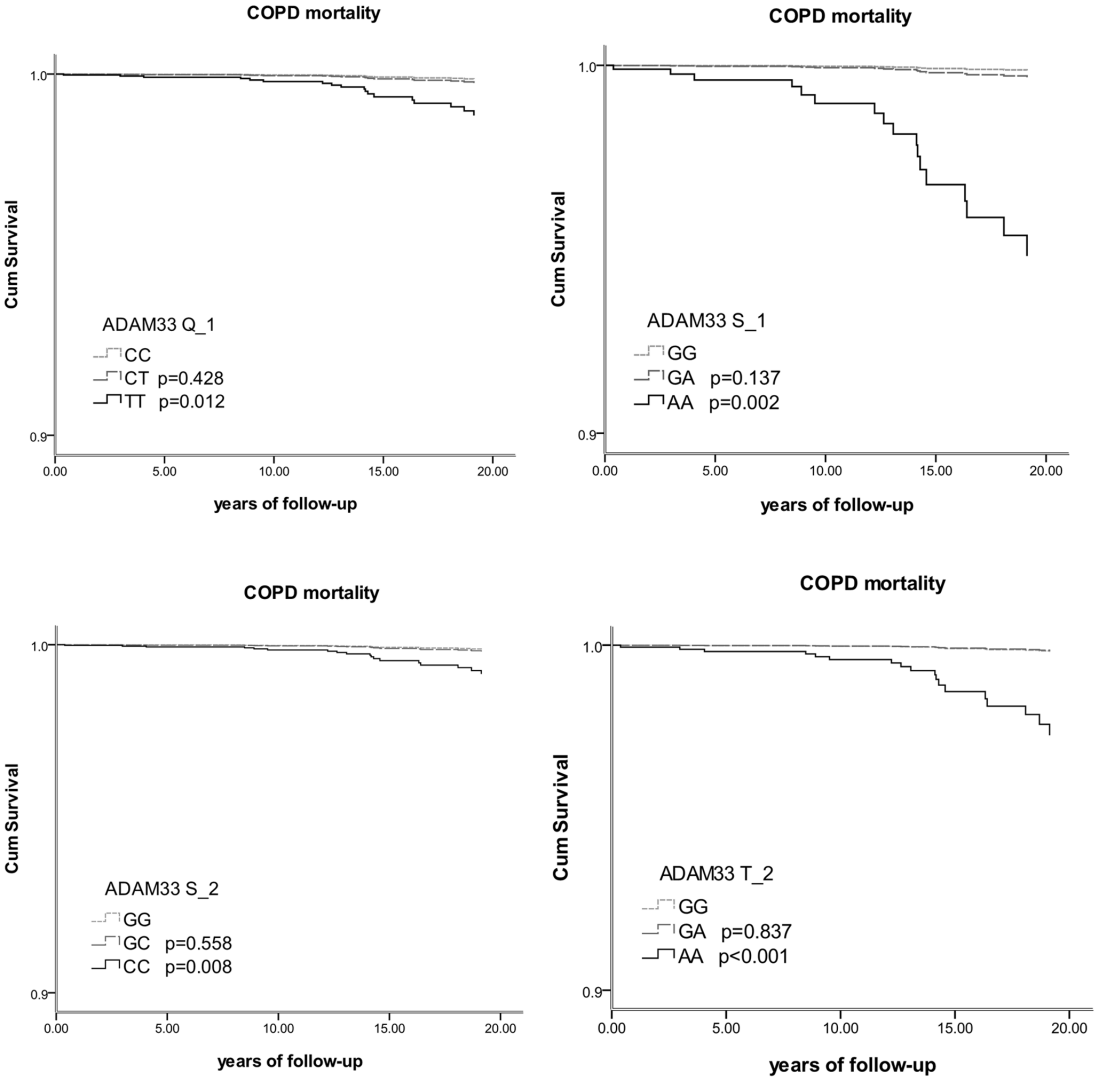
Survival curves for COPD mortality according to SNPs Q_1, S_1, S_2 and T_2.

There were significant differences in genotype distribution for SNPs Q_1, S_1, S_2 and T_2 between alive subjects and those who died due to COPD (Table 2). Carriers of minor alleles of SNPs Q_1 S_1, S_2 and T_2 had a significantly increased COPD mortality risk compared to non-carriers (Table 3). Figure 4 shows a clear trend for higher COPD mortality for subjects homozygous for SNPs Q_1 S_1, S_2 and T_2.

### Cardiovascular mortality

Individuals homozygous for the minor allele of SNP T_2 had a significantly increased risk of cardiovascular mortality compared to wild types (3.4, 1.2 to 9.5) (Table 3, Figure 3). Stratified analyses according to gender and smoking status showed that the risk of cardiovascular mortality among subjects homozygous for the minor allele of SNP T_2 was increased in all strata (all borderline significant; see Table S2). Also, females and never smokers who were homozygous for the minor allele of SNP S_1 had significantly increased cardiovascular mortality risk.

### Chance of Survival to Ages 75 and 85 yrs

At age of 75 years, subjects with the AA genotype for SNP T_2 were more likely to have died than wild types (p = 0.017, Table S3). Remarkably, none of the subjects with the AA genotype for SNP T_2 survived to the age of 85 years (Table S4).

## Discussion

The present study shows for the first time that polymorphisms in *ADAM33* are associated with all-cause, COPD and cardiovascular mortality. Thus, *ADAM33* appears to constitute an important candidate gene explaining individual differences in human lifespan. So far, *ADAM33* has been related to pulmonary diseases and lung function decline [11]–[17]. Since associations in our study are independent of lung function, these findings put a new light on the role of *ADAM33*. Of importance, effects of the SNPs were observed both in females and males, and in never and ever smokers, indicating the robustness of the associations between *ADAM33* and mortality.

*ADAM33* is preferentially expressed in smooth muscle cells, myofibroblasts, and fibroblasts [5], suggesting that this protein may be important for the functionality of the whole human organism, and likely for the lungs and the cardiovascular system.

SNP T_2 showed the broadest range of associations, since carriers of the minor alleles had an increased mortality risk for every investigated cause of death. Interestingly, the all-cause mortality risk remained significantly increased when stratified analyses were performed for gender and smoking habits. Thus SNP T_2 is associated with reduced survival, independent of other risk factors.

*ADAM33*-null mice that do not express ADAM33 at all, do not exhibit morphological or behavioral abnormalities compared to wild type mice [18]. These findings provide suggestive evidence that over-expression rather than under expression of the ADAM33 protein contributes to morbidity and in turn to mortality events.

Minor alleles of SNPs Q_1, S_1, S_2 and T_2 had a higher prevalence in subjects who died due to COPD than alive subjects, consistent with our previous findings showing a higher prevalence of the minor allele of these SNPs in subjects with COPD than in healthy controls [11]. The latter study also reported an additional association between polymorphisms in *ADAM33* and accelerated lung function decline in the general population [11]. The current study showed a higher risk of COPD mortality for individuals with polymorphisms in *ADAM33* independently of their lung function. This suggests that *ADAM33* plays a role not only in local airway events leading to impaired lung function, but also to disease progression or more extensive physiological processes which can contribute to poorer survival.

Overproduction of ADAM33 may lead to excessive shedding of inflammatory mediators and growth factors, which induce pathological states like proliferation of smooth muscle cells and fibroblasts observed in pulmonary and cardiovascular disorders [6], [11].

ADAM33 protein isoforms occur in human embryonic lungs, suggesting a role in airway development [19]. SNPs in *ADAM33* predict poor lung function in early childhood [20], thus it is plausible that ADAM33 plays a role in tissue development, and that minor alleles of the *ADAM33* SNPs lead to pathological conditions in lungs.

So far the role of *ADAM33* in cardiovascular disease is poorly understood. Holloway et al showed that *ADAM33* expression was higher in atherosclerotic lesions than in the normal vascular wall and found an association between an intronic polymorphism (rs574174 ST_7) in *ADAM33* and atherosclerosis severity [6]. Moreover ADAM12, a member of the same subfamily and closely related to ADAM33, is involved in development of cardiac hypertrophy that leads to sudden cardiac death [21].

Taking all results into account, we suggest that SNPs in *ADAM33* can be considered a risk factor for all-cause and disease specific mortality. Furthermore, since we found that subjects with the AA genotype for SNP T_2 had a lower chance to reach the age of 75, and all carriers of this genotype had died before the age of 85 we believe that the current study is an important step towards identifying genes influencing human lifespan. This may suggest that screening for this SNP, probably in conjunction with other SNPs in genes, may identify subjects who are at risk for premature death. Additionally screening for SNP T_2 may allow direct identification of subjects at risk for COPD or cardiovascular mortality. Given the increased *ADAM33* expression in smooth muscle cells in atherosclerosis [6] and following our hypothesis that overexpression of *ADAM33* may lead to the pathological events, subjects at risk may receive tailored therapy with a special target on *ADAM33* levels or activity. SNP T_2 is located in the T-exon encoding a cytoplasmic domain. In this light it is interesting to note that loss of the membrane anchor and regulatory cytoplasmic domain of *ADAM33* results in a disease-related gain of function and release from cell membrane a soluble ADAM33 form. This form in turn induces endothelial cells differentiation and promotes angiogenesis, a process important in tissue inflammation and remodeling [22]. Therefore if in subjects homozygous for the minor allele of T_2 a disease-related gain of function occurs, these may receive a special therapy. Our findings, that link *ADAM33* to the main leading diseases worldwide, reveal potential novel therapeutic targets. Hypothetically, a new drug that controls the unfavorable ADAM33 activity could prevent development of both COPD and CVD via regulation of pathological neovascularization. However to this end more studies are needed.

A major strength of this research is an excellent follow-up rate, since only 1.4% of genotyped participants could not be traced back after 18 years.

The small number of deaths due to COPD (i.e. 20) could be considered a limitation of our study. However, all SNPs which showed associations with COPD mortality were associated with COPD development in previous studies.

We decided to not correct our results for multiple testing, since our hypotheses were stated a priori and based on previous evidence indicating a role of *ADAM33* polymorphisms in pathophysiology of COPD and CVD, thus following Steiner’s advice: “If the primary outcomes have been specified beforehand, then correcting for multiplicity may be too conservative and should be avoided” [23]. Although adjustment for multiple testing will decrease the chance of type I error, it will also increase the likelihood of type II errors and potentially useful observations may be prematurely discarded [23], [24].

In summary, this study implicates that *ADAM33* is involved in all-cause mortality and in mortality due to both COPD and cardiovascular disease and these associations are independent of level of lung function, gender and smoking habits. Our findings highlight the importance of *ADAM33* as a pleiotropic gene involved not only in pulmonary disease, but in cardiovascular disease as well. Since polymorphisms in this gene are associated with increased mortality risk and with a reduced chance of survival to age of 75, we believe that *ADAM33* may affect human lifespan. Future studies should focus on the functionality of the various SNPs in this gene to further unravel its role in ageing.

## Supporting Information

Table S1 ICD-codes for the investigated causes of death.
(DOC)

Table S2 Risk of cardiovascular mortality according to gender and smoking habits.
(DOC)

Table S3 Distribution of genotypes according to being alive or dead at the age of 75, and chance of survival to this age.
(DOC)

Table S4 Distribution of genotypes according to being alive or dead at the age of 85, and chance of survival to this age.
(DOC)
