# Supplementary material for: ADAM33 Gene Polymorphisms and Mortality. A Prospective Cohort Study
Source: PLoS One. 2013 Jul 4;8(7):e67768. doi: 10.1371/journal.pone.0067768 (PMC3701578; doi:10.1371/journal.pone.0067768)
Supplement: Table S1 — ICD-codes for the investigated causes of death. (DOC) [file pone.0067768.s001.doc]

**Table S1** ICD-codes for the investigated causes of death

| Cause of death | ICD-9 | ICD-10 |
| --- | --- | --- |
| External causes* | ≥ 800 | S, T, V, W, X, Y |
| COPD | 490-492, 494, 496 | J40-J44, J47 |
| Cardiovascular disease | 390-398, 401-405, 410-417, 420-438, 440-448, 451-459, 785.4 | G45-G46, I00-I15, I20-I28, I30-I52, I60-I69, I70-I79, I80-I89, I95-I97, I98.2, I98.8, I99, M30-M31, N28.0, R02, R58 |

* Suicides, homicides*,* traffic accidents *etc.*
