# Supplementary material for: ADAM33 Gene Polymorphisms and Mortality. A Prospective Cohort Study
Source: PLoS One. 2013 Jul 4;8(7):e67768. doi: 10.1371/journal.pone.0067768 (PMC3701578; doi:10.1371/journal.pone.0067768)
Supplement: Table S2 — Risk of cardiovascular mortality according to gender and smoking habits. (DOC) [file pone.0067768.s002.doc]

**Table S2** Risk of cardiovascular mortality according to gender and smoking habits

| SNP | Genotype | Gender | | Smoking status | |
| --- | --- | --- | --- | --- | --- |
|  |  | Females | Males | Never smokers | Ever smokers |
|  |  | HR (95% CI) | HR (95% CI) | HR (95% CI) | HR (95% CI) |
| **Q_1** | CT | 0.9 (0.4-2.1) | 1.1 (0.6-2.0) | 1.1 (0.4-3.2) | 1.0 (0.6-1.8) |
|  | TT | 1.8 (0.2- 3.1) | - | 2.4 (0.3-18.6) | - |
| **S_1** | GA | 0.5 (0.1-1.5) | 0.8 (0.4-1.7) | 0.3 (0.0-2.3) | 0.8 (0.4-1.5) |
|  | AA | 10.2 (1.3-78.7)* | - | 17.4 (2.0-148.6)* | - |
| **S_2** | GC | 2.0 (1.0-4.0) | 1.2 (0.7-2.0) | 1.4 (0.6-3.4) | 1.4 (0.9-2.3) |
|  | CC | 1.6 (0.4-5.8) | 1.3 (0.5-3.7) | 1.9 (0.4-8.9) | 1.3 (0.5-3.3) |
| **T_2** | GA | 1.8 (0.9-3.6) | 1.2 (0.7-2.2) | 1.1 (0.4-3.0) | 1.5 (0.9-2.5) |
|  | AA | 4.0 (0.9-18.3) | 2.8 (0.7-11.7) | 6.5 (0.8-51.7) | 2.8 (0.9-9.3) |

**Females** n=676 (40 deaths); **Males** n=714 (67 deaths); **Never smokers** n=445 (26 deaths); **Ever smokers** n=945 (81 deaths)

***** P value < 0.05
