# Supplementary material for: ADAM33 Gene Polymorphisms and Mortality. A Prospective Cohort Study
Source: PLoS One. 2013 Jul 4;8(7):e67768. doi: 10.1371/journal.pone.0067768 (PMC3701578; doi:10.1371/journal.pone.0067768)
Supplement: Table S3 — Distribution of genotypes according to being alive or dead at the age of 75, and chance of survival to this age. (DOC) [file pone.0067768.s003.doc]

**Table S3** Distribution of genotypes according to being alive or dead at the age of 75, and chance of survival to this age

| SNP | Genotype | Dead at the age of 75 | Alive at the age of 75 | P value* | Chance of survival to age of 75  OR (95% CI) |
| --- | --- | --- | --- | --- | --- |
| **Q_1** | CC | 126 (77.3) | 202 (77.4) |  | 1 |
|  | CT | 35 (21.5) | 54 (20.7) | 0.853 | 1.1 (0.6-2.2) |
|  | TT | 2 (1.2) | 5 (1.9) |  | 2.2 (0.2-22.8) |
| **S_1** | GG | 142 (85.5) | 226 (85.3) |  | 1 |
|  | GA | 23 (13.9) | 37 (14.0) | 0.982 | 1.4 (0.6-3.1) |
|  | AA | 1 (0.6) | 2 (0.7) |  | 0.5 (0.0-6.7) |
| **S_2** | GG | 89 (54.6) | 146 (55.7) |  | 1 |
|  | GC | 57 (35.0) | 101 (38.6) | 0.190 | 1.3 (0.8-2.4) |
|  | CC | 17 (10.4) | 15 (5.7) |  | 0.7 (0.3-1.8) |
| **T_2** | GG | 111 (67.3) | 192 (75.9) |  | 1 |
|  | GA | 45 (27.3) | 58 (22.9) | 0.017 | 1.0 (0.6-1.9) |
|  | AA | 9 (5.4) | 3 (1.2) |  | 0.2 (0.0-0.9)** |

Logistic regression adjusted for gender, age, FEV_1_, height, place of residence and packyears of smoking at survey in 1989/90

* Differences between subjects alive at the age of 75 and those who died before this age tested with χ^2^ test

** P value=0.040
