# Supplementary material for: ADAM33 Gene Polymorphisms and Mortality. A Prospective Cohort Study
Source: PLoS One. 2013 Jul 4;8(7):e67768. doi: 10.1371/journal.pone.0067768 (PMC3701578; doi:10.1371/journal.pone.0067768)
Supplement: Table S4 — Distribution of genotypes according to being alive or dead at the age of 85, and chance of survival to this age. (DOC) [file pone.0067768.s004.doc]

| SNP | Genotype | Dead at the age of 85 | Alive at the age of 85 | P value* | Chance of survival to  age of 85  OR (95% CI) |
| --- | --- | --- | --- | --- | --- |
| **Q_1** | CC | 197 (77.9) | 44 (75.9) |  | 1 |
|  | CT | 52 (20.6) | 13 (22.4) | 0.947 | 1.2 (0.4-3.1) |
|  | TT | 4 (1.6) | 1 (1.7) |  | 1.0 (0.1-15.3) |
| **S_1** | GG | 222 (86.0) | 49 (81.7) |  | 1 |
|  | GA | 34 (13.2) | 11 (18.3) | 0.475 | 2.2 (0.8-6.5) |
|  | AA | 2 (0.8) | 0 (0.0) |  | - |
| **S_2** | GG | 138 (54.1) | 36 (61.0) |  | 1 |
|  | GC | 96 (37.6) | 19 (32.2) | 0.629 | 0.9 (0.4-2.2) |
|  | CC | 21 (8.2) | 4 (6.8) |  | 1.1 (0.2-6.2) |
| **T_2** | GG | 174 (68.2) | 44 (78.6) |  | 1 |
|  | GA | 70 (27.5) | 12 (21.4) | 0.154 | 1.1 (0.4-2.8) |
|  | AA | 11 (4.3) | 0 (0.0) |  | - |

**Table S4** Distribution of genotypes according to being alive or dead at the age of 85, and chance of survival to this age

Logistic regression adjusted for gender, age, FEV_1_, height, place of residence and packyears of smoking at survey in 1989/90

* Differences between subjects alive at the age of 85 and those who died before this age tested with χ^2^ test
